# Presence and Dermal Exposure to Benzene and Acetaldehyde in Hand Sanitizers Available in Taiwan

**DOI:** 10.3390/toxics13070537

**Published:** 2025-06-26

**Authors:** Chieh-An Cheng, Shih-Wei Tsai

**Affiliations:** Institute of Environmental and Occupational Health Sciences, College of Public Health, National Taiwan University, No. 17, Xuzhou Road, Taipei 100, Taiwan

**Keywords:** COVID-19, alcohol-based hand sanitizers, impurities, health risk assessment

## Abstract

The widespread use of alcohol-based hand sanitizers (ABHS) during and after the COVID-19 pandemic has raised concerns about potential exposure to harmful volatile organic compounds (VOCs), such as benzene, acetaldehyde, and other impurities, which may pose health risks. This study investigated the concentrations of ethanol, isopropanol, and 12 impurities, including benzene, acetaldehyde, and methanol, in 85 commercially available ABHS products in Taiwan using gas chromatography-mass spectrometry (GC-MS). The results revealed that 12 samples contained alcohol concentrations below the recommended 60% (*v*/*v*) threshold. Benzene and acetaldehyde were identified as the primary impurities, with mean concentrations of 0.84 μg/g and 22.39 μg/g, respectively, exceeding the US FDA interim limits. For frequent ABHS users, the average dermal exposure doses (DEDs) to benzene ranged from 3.17 × 10^−2^ to 15.5 μg/kg-bw/day, with children aged 2–11 years showing the highest non-carcinogenic risk (Hazard Quotient > 1) and cancer risk (6.37 × 10^−5^ to 9.33 × 10^−4^). The findings emphasize the need for stringent quality control of ABHS products and caution in their selection and use. This study provides critical insights into the health risks associated with ABHS in Taiwan, underscoring the importance of regulatory oversight to ensure consumer safety.

## 1. Introduction

During the COVID-19 pandemic, the Centers for Disease Control and Prevention (CDC) in the United States has advocated for the utilization of face masks, disinfection, and proper hand hygiene as effective measures to mitigate the spread of the virus [1]. Hand sanitizers have been identified as a practical method to interrupt virus transmission through direct and indirect contact when handwashing with soap and water is not readily available [2,3]. In addition to the US CDC, other organizations such as the World Health Organization (WHO) and the US Food and Drug Administration (FDA) have endorsed alcohol-based hand sanitizers (ABHS) as an alternative hand hygiene practice [4].

Commercially available hand sanitizers come in various forms, including liquid, gel, foam, cream, and wipes. They are formulated with different ingredients, such as antimicrobial agents, alcohol-free compounds, or alcohol-based solutions [5]. Various organizations have established guidelines regarding the formulation of alcohol-based hand sanitizers. For instance, the US CDC recommends a minimum alcohol concentration of 60% in ABHS, as ethanol levels below this threshold may not effectively disrupt microbial cell membranes. Conversely, the WHO suggests an alcohol concentration range of 60–95% and offers two formulations: one containing 80% ethanol and the other with 75% isopropanol [5,6].

To effectively utilize ABHS, it is advisable to thoroughly rub the hands until they are completely dry. Research has indicated that for effective virus eradication, a single application of hand sanitizer should range from 1 mL to 3 mL, with the US Food and Drug Administration (US FDA) recommending 2.4 mL as an appropriate volume [7]. The Centers for Disease Control in Taiwan (Taiwan CDC) has recommended using a minimum of 2 to 3 mL of ABHS during hand hygiene practices, ensuring a duration of 20 to 30 s for the hands to dry.

Nevertheless, the US FDA cautioned consumers against using specific hand sanitizers from certain manufacturers in June 2020 due to the presence of methanol instead of ethanol [8]. The US FDA continued to provide guidance on detecting impurities, advised consumers to scrutinize hand sanitizer labels and packaging, and issued a list of hand sanitizers to avoid. Notably, some commercially available ABHS lacked the requisite ethanol content, and certain impurities such as methanol, 1-propanol, benzene, acetaldehyde, and acetal were identified.

With the widespread use of ABHS globally, regulatory health agencies such as the US FDA, Health Canada, and Agência Nacional de Vigilância Sanitária (ANVISA) have adapted their guidelines to address the increasing demand for hand sanitizers. However, concerns have been raised regarding the quality of commercially available ABHS. A study in Brazil examined 48 gel ABHS products from drugstores and supermarkets, revealing ethanol content ranging from 43.9% to 77.3% (*w*/*w*), with 13 products failing in at least one category related to ethanol content. Additionally, qualitative analysis identified ethyl acetate and acetaldehyde in 12 samples [2].

Another study in Durban, KwaZulu-Natal, South Africa, analyzed 50 hand sanitizers, finding alcohol content ranging from 44% to 93%, with 32% not meeting regulations and 16 samples failing to meet labeling requirements [9]. Furthermore, three ABHS samples were found to contain 1-propanol and ethyl acetate as contaminants [9]. Tse et al. examined 42 ABHS products (26 liquid and 16 gelled) in Canada, discovering that 11 samples did not meet Health Canada interim guidelines, with a maximum acetaldehyde concentration of 251 ± 10 μL/L (3.3 times higher than the current standard) [3]. Moreover, the total acetal and acetaldehyde concentration in 17 samples exceeded the levels specified in the United States Pharmacopeia (USP) monograph, while ethanol content in 26 liquid ABHS samples ranged from 63% to 90% (*v*/*v*) [3].

A study in Bangladesh analyzed approximately 200 samples, revealing that 28 were contaminated with methanol [6]. The American Association of Poison Control Centers (AAPCC) estimated around 8000 reported cases of exposure to hand sanitizers or disinfectants between January 2017 and May 2021 [10]. Ethanol and isopropanol are the primary ingredients in sanitizers, but impurities can have adverse effects on human health, particularly causing skin reactions.

The most common skin reactions include irritant contact dermatitis (ICD) and allergic contact dermatitis (ACD), which can manifest as itching, dryness, bleeding, erythema, and, in severe cases, anaphylactic symptoms or respiratory distress [11,12,13,14]. While ethanol and isopropanol, the main ABHS ingredients, are less irritating to the skin compared to 1-propanol, they can still lead to skin dryness, flaking, or cracking [15,16,17]. Impurities such as methanol can cause ocular disturbances and blindness with repeated skin contact [18]. Acetaldehyde irritates various body parts and can induce symptoms such as nausea, vomiting, headache, dermatitis, and pulmonary edema. Ethyl acetate may cause dry or cracked skin upon repeated contact and could have lasting impacts on the liver and kidneys (New Jersey Department of Health and Senior Services).

To mitigate the aforementioned adverse health effects, it is recommended to select products with fewer irritating ingredients, utilize moisturizing creams that contain humectants, fats, or oils to preserve skin moisture, and take into account environmental variables such as temperature and humidity, which can influence skin hydration levels [19,20,21].

To the best of our knowledge, in Taiwan, ABHS products containing ethanol and isopropanol have been predominantly utilized over alcohol-free alternatives. However, there is a lack of information regarding the concentrations of alcohols and impurities present in these ABHS products. Previous research has indicated the presence of impurities in certain products, which could potentially pose health hazards upon prolonged exposure. Furthermore, ABHS products with insufficient levels of ethanol or isopropanol (ranging from 60–90%) may compromise their effectiveness in disinfection. Due to the widespread use of personal disinfectants in Taiwan following the COVID-19 pandemic, it is crucial to assess the potential health risks associated with ABHS use. Therefore, the aim of this study was to examine the levels of ethanol, isopropanol, and twelve impurities in ABHS products, including methanol, acetaldehyde, acetone, ethyl acetate, isobutanol, benzene, acetal, 1-propanol, 2-butanol, 1-butanol, 3-methyl-1-butanol, and amyl alcohol, and to assess the health risks associated with exposure to these identified chemicals.

## 2. Experimental

### 2.1. Reagents and Chemicals

Fourteen specific chemicals were employed in this study, including ethanol, isopropanol, methanol, acetone, ethyl acetate, 1-propanol, 1-butanol, 2-butanol, isobutanol, acetal, 3-methyl-1-butanol, amyl alcohol, benzene, and acetaldehyde. Ethanol was procured from Dr. Ehrenstorfer™, while isobutanol was obtained from J.T.Baker. Methanol, ethyl acetate, acetal, acetone, benzene, acetaldehyde, and methyl propyl ketone (MPK) were obtained from Sigma-Aldrich and served as internal standards in the study. Isopropanol, 1-butanol, 2-butanol, 3-methyl-1-butanol, and amyl alcohol were acquired from Tokyo Chemical Industry Co., Ltd. (Tokyo, Japan). Additionally, acetone-d6 was selected as a surrogate and was purchased from ThermoFisher.

### 2.2. Sample Collection and Preparation

A total of 85 alcohol-based hand sanitizer (ABHS) products were collected from the Taiwanese market between May and July 2022. The samples were obtained from a combination of retail pharmacies, convenience stores, supermarkets, and online platforms, reflecting common consumer purchasing channels. Each sample corresponded to a different brand or formulation. All products were labeled as either containing ethanol or isopropanol as the primary active ingredient, and no products required a prescription for purchase. These products were categorized into two primary groups based on their physical form: liquid (N = 53) and gel (N = 32). The entire set of 85 samples was subjected to analysis within the scope of this research.

In the process of sample preparation, approximately 0.1 g of each product was introduced into a 10 mL volumetric flask, followed by the addition of solvent, specifically Milli-Q water, to reach a total volume of 10 mL. Subsequently, the mixture was vortexed for a duration of 3 min to ensure thorough homogenization. To ascertain the efficacy of this procedure, a known surrogate concentration was incorporated to determine the recovery rate. The samples were then stored in vials of either 15 mL or 20 mL capacity, sealed with parafilm, and maintained at a temperature of 4 °C until the time of analysis. The extent of dilution varied depending on the specific target chemicals being analyzed. For instance, ethanol and isopropanol were subjected to a serial dilution ratio of 1:37,500, whereas other target chemicals were diluted at a ratio of 1:1000. The number of dilutions performed on the samples was contingent upon whether the concentrations exceeded the ranges of the calibration curve.

A 1 μL aliquot of the final diluted sample was injected directly into the GC-MS system without further cleanup or concentration. This is because the analytes of interest (e.g., benzene, acetaldehyde, methanol) are volatile, low-molecular-weight compounds that are either pre-dissolved in the alcohol matrix or readily soluble in the aqueous–alcohol solution. Therefore, no extraction or pre-treatment step was required. This direct-injection approach is consistent with established analytical procedures for ABHS analysis (e.g., USP <467>, NIST IR 8342) and provides sufficient sensitivity and specificity for the target compounds [22].

### 2.3. Instrumentation

The analysis by gas chromatography/mass spectrometer (GC-MS) was conducted utilizing an Agilent 6890N gas chromatograph connected to an Agilent 7001B mass spectrometer (Agilent Technologies, Santa Clara, CA, USA). A capillary column (Rtx-Volatile, dimensions 60 m × 0.32 mm × 4.5 μm film thickness) was employed in the investigation. Helium gas of 99.999% purity was used as the carrier gas at a flow rate of 1 mL/min. The injector temperature for most target chemicals, such as ethanol, isopropanol, methanol, acetone, ethyl acetate, isobutanol, benzene, acetal 1-propanol, 2-butanol, 1-butanol, 3-methyl-1-butanol, and amyl alcohol, was set at 235 °C with pulsed split mode (20:1).

The GC oven program was initiated at 80 °C for 2 min with a ramp rate of 8 °C/min, then increased to 120 °C (for 0.5 min), followed by a further increase to 180 °C at 10 °C/min (for 0.5 min), reaching 240 °C at 15 °C/min (for 2 min), and finally concluding at 260 °C (for 2 min) in the post-run phase. An alternate oven program was utilized for acetaldehyde, particularly with an injector temperature of 220 °C. The GC oven program for acetaldehyde began at 40 °C for 5 min with a ramp rate of 8 °C/min, then elevated to 140 °C, and subsequently increased to 220 °C at 10 °C/min (5 min), concluding at 260 °C (for 2 min) in the post-run phase.

Mass spectrometry was conducted in electron impact (EI) mode at 70 eV, with the transfer line temperature set at 220 °C and the ion source at 230 °C. Selected ion mode (SIM) was employed for target chemicals and scan mode for other potential impurities, utilizing the NIST and Wiley library database for qualitative analysis. The quantification ions for each target compound were selected based on their most abundant and specific fragment ions in the SIM (selected ion monitoring) mode, typically corresponding to the highest-intensity *m*/*z* values in the mass spectrum. A gain factor of 1 was applied, and the scan cycles for all time segments were maintained above three cycles per second. The retention time (RT) and selected ions for the analysis are detailed in Table 1.

### 2.4. Method Validation

The primary method involved combining a standard solution with deionized water and mixing benzene with methanol to generate a calibration curve (Table 1). Method precision was evaluated by conducting triplicate measurements at both the lower and upper ends of the calibration curve to determine the relative standard deviation (RSD). Acetone-d6 was used as a surrogate standard to determine the recovery rate. In addition, the method detection limits (MDLs) were determined using the calculation procedures suggested by the US EPA (where MDL = standard deviation of replicate analyses × Student’s t-value for the 99% confidence level with n − 1 degrees of freedom) (US EPA).

Limit of quantification (LOQ) was determined based on a signal-to-noise (S/N) ratio of 10:1 using spiked matrix samples at low concentrations. The LOQ values for the target analytes were as follows: benzene (0.4 μg/g), acetaldehyde (0.9 μg/g), methanol (0.5 μg/g), ethanol (0.3 μg/g), and isopropanol (0.3 μg/g). Certified reference standards with purities >99.5% were purchased from Sigma-Aldrich and used to prepare calibration solutions. Recovery and accuracy were assessed by spiking blank matrix samples at three levels (low, mid, and high). Mean recoveries ranged from 94.1% to 104.6%.

### 2.5. Dermal Exposure Assessment

An assessment of dermal exposure was carried out for the specified impurities based on the analytical findings. The risk estimation in this investigation utilized a formula recommended by the US Environmental Protection Agency [23] as cited in the work by Pal et al. [24]. The dermal exposure dose (DED) was calculated using the formula:

$$\mathrm{DED}=\frac{C\times A}{BW}$$

where DED represents the dermal exposure dose in micrograms per kilogram of body weight per day (μg/kg-bw/day), *C* is the concentration of the target chemical in micrograms per gram (μg/g), A is the applied amount of the product in grams per day, and BW is the body weight in kilograms.

Among the target chemicals, benzene was categorized as a Group 1 human carcinogen by the International Agency for Research on Cancer [25], thus necessitating assessment for non-carcinogenic (hazard quotient; HQ) and carcinogenic risks (CR). The relevant formulas are as follows:

$$\mathrm{HQ}=\frac{DED}{RfD};\;\mathrm{CR}=\mathrm{DED}\times \mathrm{DSF}$$

where RfD is the reference dose in micrograms per kilogram of body weight per day, specifically 4 μg/kg-bw/day for benzene. An HQ value exceeding one indicates a potential for adverse effects on human health. DSF is the dermal cancer slope factor in kilograms per day per microgram, set at 5 × 10^−5^ for benzene. A CR value surpassing the acceptable threshold signifies a significant cancer risk.

Moreover, in cases where data exhibited high skewness (geometric standard deviation (GSD) exceeding 3), non-detected values of the target chemicals were substituted with a representative value of $\frac{LOD}{\sqrt{2}}$ as suggested by Hornung and Reed [26].

## 3. Results and Discussions

### 3.1. Analysis with GC-MS

**Figure 1:**
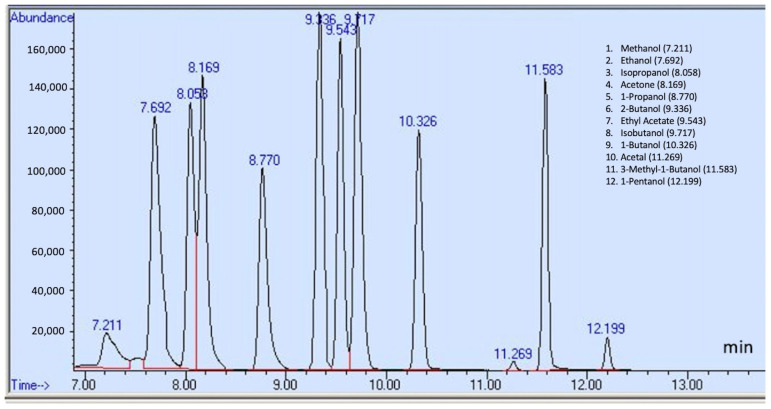
Chromatogram of the 12 target compounds in SIM mode.

**Figure 2:**
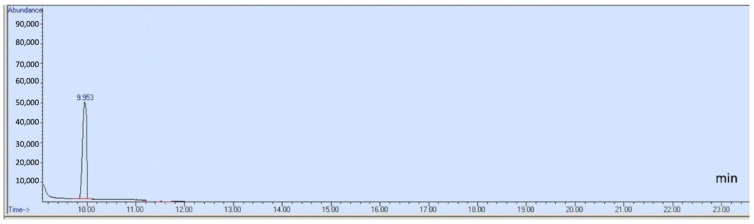
Chromatogram of acetaldehyde in SIM mode.

**Figure 3:**
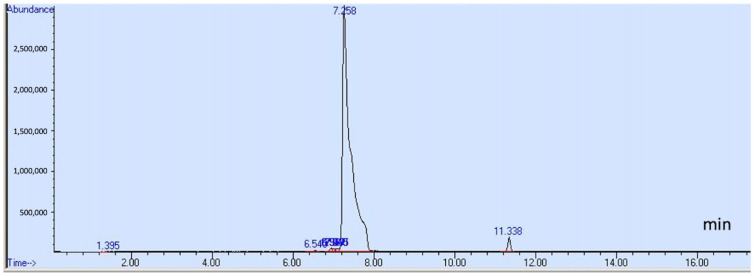
Chromatogram of benzene (11.338) in SIM mode.

This study individually examined acetaldehyde, benzene, and other specified compounds due to their distinct chemical properties. A 20:1 split ratio was employed in selected ion monitoring (SIM) mode to improve the resolution and sharpness of the compound peaks. Figure 1 displays the total ion chromatogram (TIC) of 12 compounds, which include methanol, ethanol, isopropanol, acetone, 1-propanol, 2-butanol, ethyl acetate, isobutanol, 1-butanol, acetal, 3-methyl-1-butanol, and 1-pentanol. The chromatograms for acetaldehyde and benzene are presented in Figure 2 and Figure 3, respectively. Table 1 details each analyte’s retention time, ion quantification and identification, and calibration ranges.

Table 1 lists the method detection limits (MDLs) and relative standard deviations (RSDs) established in this study. The MDLs range from 0.0017 to 3.74 μg/mL, while the RSD ranges from 0.93% to 12.68%.

Although GC-MS served as the principal analytical platform in this study, its performance was fully validated in-house in accordance with USP <467>. According to NIST IR 8342, GC-MS offers the highest selectivity and sensitivity among available platforms, allowing for definitive identification and quantification of regulated impurities such as benzene and acetaldehyde at sub-ppm levels [22]. Because GC-MS remains the reference technique cited in current FDA surveillance guidelines, our results are comparable with international monitoring data and are sufficient for the exposure assessment presented here.

### 3.2. Sample Analysis

In the majority of samples analyzed, ethanol was present, while isopropanol was only detected in two samples at concentrations of 42% and 58%. The World Health Organization (WHO) recommends an alcohol concentration ranging from 60 to 90% (*v*/*v*); however, this investigation identified 12 products that did not adhere to this standard. Despite being labeled as containing 75% alcohol, discrepancies between the stated content and actual composition were observed in the study. The findings suggest the necessity for regulatory bodies to enforce the inclusion of quality inspection reports on the packaging of alcohol-based hand sanitizers. Furthermore, manufacturers are encouraged to disclose product ingredients accurately on labels to ensure consumers are informed about their effectiveness.

As shown in Table 2, the results revealed that the mean concentrations of benzene were 0.84 μg/g, ranging from 1.57 to 33.99 μg/g, while acetaldehyde levels averaged at 22.39 μg/g, with a range of 30.09–429.04 μg/g, exceeding the interim limit specified in the US FDA guidance, necessitating immediate attention. According to the USP general chapter <467> Residual Solvents [27], benzene is categorized as a class 1 impurity, and methanol is classified under class 2. Acetone, 1-propanol, ethyl acetate, 2-butanol, isobutanol, 1-butanol, 3-methyl-1-butanol, and amyl alcohol are grouped as class 3 impurities. It is advisable to eliminate class 1 solvents, restrict class 2 solvents, and acknowledge that class 3 solvents pose comparatively lower risks. Consequently, the impurities identified in this study should be treated with seriousness.

Moreover, acetal has the potential to serve as a precursor for acetaldehyde, particularly in acidic environments, making it more conducive to acetaldehyde formation. Conversely, under primary conditions, acetal is more likely to be formed [28,29]. According to a previous study, the pH range of hand sanitizer samples was 3.1–8.4 [30], indicating that the presence of acetaldehyde often precludes the detection of acetal in the majority of cases.

To contextualize the findings of this study, the results for benzene and acetaldehyde were compared with international surveillance studies. In this Taiwan-based survey, benzene was detected in 5 of 85 samples (5.9%), with a maximum concentration of 33.99 μg/g and a median of 1.57 μg/g (Table 2). The detection rate was comparable to that reported by the U.S. National Institute of Standards and Technology (NIST), which found benzene in 6 of 72 samples (8.3%) [22], and in a petition by Valisure LLC, which identified benzene above the interim limit in approximately 8% of 260 hand sanitizers [31]. However, the maximum benzene concentration in the present study was lower than the highest levels found in the U.S., where individual samples reportedly exceeded 80 μg/g.

In contrast, acetaldehyde was detected in 19 of 85 samples (22.4%), with a median concentration of 54.67 μg/g and a maximum of 429.03 μg/g (Table 2). While the detection rate was lower than that observed in an international survey by Saab et al. [32], which reported benzene detection in 58% of products but did not quantify acetaldehyde, the concentrations in the Taiwan samples were comparable to or higher than levels of concern reported elsewhere. Notably, the median acetaldehyde level in this study exceeded the FDA’s provisional impurity threshold of 50 μg/g, indicating that a substantial portion of local products may not meet international quality standards.

These findings suggest that although the occurrence of benzene in Taiwan ABHS products appears limited and aligns with other markets, acetaldehyde contamination may pose a greater regulatory concern due to its elevated concentration, even in fewer detections.

### 3.3. Estimations of Exposures

Numerous prior investigations have demonstrated a notable surge in the utilization of hand sanitizers within the general populace subsequent to the onset of the COVID-19 pandemic. The examination of children in educational or childcare environments determined that individuals aged 4–17 years tended to employ hand sanitizers most frequently at a rate of 4–6 times per day [33]. Specifically, 21% of children reported utilizing hand sanitizers 1–3 times daily, while fewer than 5% disclosed using them 7–9 or 10–14 times daily [33]. Consequently, the frequency of usage was categorized into three distinct groups: low (2 times/day), moderate (6 times/day), and high (9 times/day) in this study.

Considering the variance in behavior across different age groups and referencing the Exposure Dose Guidance for Body Weight provided by the Agency for Toxic Substances and Disease Registry (ATSDR), body weight was segmented into three categories: 24.6 kg (2–11 years old), 64.2 kg (11–21 years old), and 80 kg (≥21 years old) [33,34,35]. Despite the existing recommendations on usage quantities by various countries and organizations, this study and associated research have revealed that the average application volume is 1.5 mL per use for liquid formulations and 1.5 g per use for gel formulations [35].

The estimated dermal exposure doses (DEDs) of the target impurities are presented in Supplementary Table S1. The mean concentrations of DED of the target impurities ranged from 3.17 × 10^−2^ for benzene to 15.5 μg/kg-bw/day for acetal, and the geometric mean concentrations ranged from 2.88 × 10^−4^ for benzene to 2.15 μg/kg-bw/day for methanol. Furthermore, the non-carcinogenic and carcinogenic risks of dermal exposure results for benzene are presented in Table 3, respectively. 

For non-carcinogenic risks, if the calculated hazard quotient (HQ) for a specific adverse effect exceeds 1, it may indicate a potential health risk. For cancer risk (CR), if it exceeds 1.0 × 10^−6^, it might be considered to pose a potential health risk. According to the data in Table 3, the age range of 2 to 11 years old has the highest non-cancer risk value, with a maximum value ranging from approximately 1.04 to 4.66 for benzene exposure. In addition, the risk of cancer from exposure to benzene, as also shown in Table 3, is highest in the age group of 2 to 11 years when calculated with the maximum possible exposure level, ranging from 2.07 × 10^−4^ to 9.33 × 10^−4^. For other age groups, when considering their maximum possible exposure level, the cancer risk is also greater than 10^−6^. In comparison to Pal’s research, where the average benzene concentration was 395 ng/g (range: 8.1 × 10^−2^–2.23 × 10^4^) and the mean cancer risk was 1.23 × 10^−5^ (range: 2.53 × 10^−10^–6.97 × 10^−5^) [24], the findings of this study suggest that the samples obtained from the Taiwanese market could pose potential health risks.

## 4. Conclusions

This research involved collecting 85 different alcohol-based hand sanitizer products from the market, followed by analyzing their alcohol contents and impurities using GC-MS. Additionally, a dermal exposure assessment was conducted to identify impurities in the samples and highlight potential health risks associated with dermal contact. Although the concentrations of these impurities were below US FDA regulations, frequent or excessive use could still pose health concerns. Moreover, a significant number of products did not meet the minimum requirement of containing at least 60% (*v*/*v*) alcohol, which raises doubts about their disinfection effectiveness. Furthermore, several samples falsely claimed to contain 75% alcohol.

Given the possibility of encountering other viruses or bacteria in the post-epidemic period, caution is advised regarding the use of alcohol-based hand sanitizers, especially in settings like hospitals, healthcare facilities, schools, and daycares. The study recommended periodic government inspections of market products to safeguard consumer safety and health. It also suggested that the general public exercise caution when using these products and consider washing hands with tap water as an alternative.

## Figures and Tables

**Table 1:** GC/MS parameters, calibration ranges, MDLs, RSDs, and US FDA’s interim limits.

Compounds	Retention Time (min)	Quantification Ion (*m*/*z*)	Identification Ions (*m*/*z*)	Calibration Range (μg/mL)	MDLs (μg/mL)	RSD (%) Low Conc	RSD (%) High Conc	Interim Limit Listed in FDA Guidance (ppm) ^a, b^
Methanol	7.211	31	32, 29	20–200	3.74	8.41	2.56	NMT 630
Ethanol	7.692	31	45, 29	20–200	2.41	1.24	2.20	
Isopropanol	8.058	45	43, 27	20–200	2.57	7.27	2.89	
Acetaldehyde	9.953	29	44, 43	0.5–5	0.0017	3.48	0.93	NMT 50
Benzene	11.338	78	77, 51	0.5–1	0.0077	9.85	1.31	NMT 2
Acetone	8.169	43	58, 15	0.5–5	0.0231	10.79	7.93	NMT 4400
Ethyl acetate	9.543	43	61, 45	0.5–5	0.0334	1.25	1.75	NMT 2200
Isobutanol	9.717	43	41, 42	0.5–5	0.0346	2.04	3.20	NMT 21,700
Acetal	11.269	45	73, 29	0.5–5	0.0151	4.05	8.82	NMT 50
1-propanol	8.770	31	29, 59	0.5–5	0.0221	1.73	2.77	NMT 1000
1-butanol	10.326	56	31, 41	0.5–5	0.028	4.51	4.49	NMT 1000
2-butanol	9.336	45	27, 59	0.5–5	0.0389	9.96	2.74	NMT 6200
3-methyl-1-butanol	11.583	55	42, 43	0.5–5	0.0152	9.7	12.68	NMT 4100
1-pentanol	12.199	42	55, 41	0.5–5	0.0283	2.90	3.68	NMT 4100

^a^ NMT: Not more than. ^b^: Methanol, acetaldehyde and benzene have their own individual limits. In addition, the sum of other impurities should not exceed 300 ppm. If the total of other impurities is over 300, then each impurity should meet the regulation above.

**Table 2:** Concentrations of target compounds detected in samples (μg/g).

Chemical	Methanol	Acetone	1-Propanol	2-Butanol	Ethyl Acetate	Isobutanol	1-Butanol	Acetal	3-Methyl-1-butanol	1-Pentanol	Benzene	Acetaldehyde
detection rates * (%)	11 of 85 (12.9)	11 of 85 (12.9)	13 of 85 (15.3)	14 of 85 (16.5)	16 of 85 (18.8)	7 of 85 (8.2)	10 of 85 (11.8)	2 of 85 (2.4)	12 of 85 (14.1)	2 of 85 (2.3)	5 of 85 (5.9)	19 of 85 (22.4)
Mean **	14.61	4.53	6.73	13.83	26.34	7.24	10.23	28.24	4.65	1.32	0.84	22.39
GM ***	3.91	0.05	0.05	0.09	0.11	0.05	0.05	0.07	0.03	0.02	0.01	0.01
Max	318.99	61.32	105.68	365.07	433.25	167.56	217.54	387.57	73.12	72.82	33.99	429.03
Min	7.15	7.92	2.85	0.13	21.18	31.78	12.71	23.21	13.96	37.69	1.57	30.09
Median	50.74	25.96	39.57	29.64	99.45	86.96	53.11	114.66	28.95	55.26	4.19	54.67

* detection rates: the impurities which were detected among all products (%). ** samples which were not detected were calculated with the value of the detection limit divided by the square root of 2. *** GM: geometric mean.

**Table 3:** The non-carcinogenic and cancer risks from dermal exposure to benzene.

The non-carcinogenic risks									
	Application amount (3 g/day) Body weight (24.6 kg) 2~11 years old	Application amount (3 g/day) Body weight (64.2 kg) 11~21 years old	Application amount (3 g/day) Body weight (80 kg) >21 years old	Application amount (9 g/day) Body weight (24.6 kg) 2~11 years old	Application amount (9 g/day) Body weight (64.2 kg) 11~21 years old	Application amount (9 g/day) Body weight (80 kg) >21 years old	Application amount (13.5 g/day) Body weight (24.6 kg) 2~11 years old	Application amount (13.5 g/day) Body weight (64.2 kg) 11~21 years old	Application amount (13.5 g/day) Body weight (80 kg) >21 years old
Mean	2.57 × 10^−2^	9.86 × 10^−3^	7.92 × 10^−3^	7.72 × 10^−2^	2.96 × 10^−2^	2.37 × 10^−2^	1.16 × 10^−1^	4.44 × 10^−2^	3.56 × 10^−2^
GM	2.34 × 10^−4^	8.98 × 10^−5^	7.21 × 10^−5^	7.03 × 10^−4^	2.69 × 10^−4^	2.16 × 10^−4^	1.05 × 10^−3^	4.04 × 10^−4^	3.24 × 10^−4^
Max	1.04 × 10^0^	3.97 × 10^−1^	3.19 × 10^−1^	3.11 × 10^0^	1.19 × 10^0^	9.56 × 10^−1^	4.66 × 10^0^	1.79 × 10^0^	1.43 × 10^0^
min	1.52 × 10^−4^	5.84 × 10^−5^	4.69 × 10^−5^	4.57 × 10^−4^	1.75 × 10^−4^	1.41 × 10^−4^	6.86 × 10^−4^	2.63 × 10^−4^	2.11 × 10^−4^
Median	1.52 × 10^−4^	5.84 × 10^−5^	4.69 × 10^−5^	4.57 × 10^−4^	1.75 × 10^−4^	1.41 × 10^−4^	6.86 × 10^−4^	2.63 × 10^−4^	2.11 × 10^−4^
The cancer risks									
	Application amount (3 g/day) Body weight (24.6 kg) 2~11 years old	Application amount (3 g/day) Body weight (64.2 kg) 11~21 years old	Application amount (3 g/day) Body weight (80 kg) >21 years old	Application amount (9 g/day) Body weight (24.6 kg) 2~11 years old	Application amount (9 g/day) Body weight (64.2 kg) 11~21 years old	Application amount (9 g/day) Body weight (80 kg) >21 years old	Application amount (13.5 g/day) Body weight (24.6 kg) 2~11 years old	Application amount (13.5 g/day) Body weight (64.2 kg) 11~21 years old	Application amount (13.5 g/day) Body weight (80 kg) >21 years old
Mean	5.15 × 10^−6^	1.97 × 10^−6^	1.58 × 10^−6^	1.54 × 10^−5^	5.92 × 10^−6^	4.75 × 10^−6^	2.32 × 10^−5^	8.88 × 10^−6^	7.12 × 10^−6^
GM	4.69 × 10^−8^	1.80 × 10^−8^	1.44 × 10^−8^	1.41 × 10^−7^	5.39 × 10^−8^	4.32 × 10^−8^	2.11 × 10^−7^	8.08 × 10^−8^	6.49 × 10^−8^
Max	2.07 × 10^−4^	7.94 × 10^−5^	6.37 × 10^−5^	6.22 × 10^−4^	2.38 × 10^−4^	1.91 × 10^−4^	9.33 × 10^−4^	3.57 × 10^−4^	2.87 × 10^−4^
min	3.05 × 10^−8^	1.17 × 10^−8^	9.38 × 10^−9^	9.15 × 10^−8^	3.50 × 10^−8^	2.81 × 10^−8^	1.37 × 10^−7^	5.26 × 10^−8^	4.22 × 10^−8^
Median	3.05 × 10^−8^	1.17 × 10^−8^	9.38 × 10^−9^	9.15 × 10^−8^	3.50 × 10^−8^	2.81 × 10^−8^	1.37 × 10^−7^	5.26 × 10^−8^	4.22 × 10^−8^

## Data Availability

The authors declare that the data supporting the findings of this study are available within the paper. Should any raw data files be needed in another format, they are available from the corresponding author upon reasonable request.

## Supplementary Materials

The following supporting information can be downloaded at: https://www.mdpi.com/article/10.3390/toxics13070537/s1, Table S1: The estimated dermal exposure assessment of the target impurities by alcohol based hand sanitizers (μg/kg-bw/day).
