# Supplementary material for: Presence and Dermal Exposure to Benzene and Acetaldehyde in Hand Sanitizers Available in Taiwan
Source: Toxics. 2025 Jun 26;13(7):537. doi: 10.3390/toxics13070537 (PMC12301027; doi:10.3390/toxics13070537)
Supplement: Supplementary file 1 [file toxics-13-00537-s001.zip › toxics-3673467-supplementary.pdf]

Supplementary Table S1. The estimated dermal exposure assessment of the target impurities by alcohol based hand sanitizers (μg/kg-bw/day)

		methanol	acetone	1-propanol	2-butanol	Ethyl Acetate	isobutanol	1-butanol	acetal	3-methyl-1-butanol	1-pentanol	benzene	acetaldehyde
Application amount	Mean	1.78E+00	5.52E-01	8.21E-01	1.69E+00	3.21E+00	8.83E-01	1.25E+00	3.44E+00	5.67E-01	1.61E-01	1.03E-01	2.73E+00
(3g/day)	GM	4.77E-01	5.58E-03	6.11E-03	1.04E-02	1.40E-02	5.67E-03	6.22E-03	8.38E-03	4.09E-03	2.94E-03	9.38E-04	1.50E-03
Body weight	Max	3.89E+01	7.48E+00	1.29E+01	4.45E+01	5.28E+01	2.04E+01	2.65E+01	4.73E+01	8.92E+00	8.88E+00	4.14E+00	5.23E+01
(24.6 kg)	min	3.22E-01	1.95E-03	1.95E-03	3.41E-03	2.93E-03	2.93E-03	2.44E-03	1.34E-03	1.34E-03	2.44E-03	6.10E-04	1.22E-04
2~11 years old	Median	3.22E-01	1.95E-03	1.95E-03	3.41E-03	2.93E-03	2.93E-03	2.44E-03	1.34E-03	1.34E-03	2.44E-03	6.10E-04	1.22E-04
Application amount	Mean	6.83E-01	2.12E-01	3.14E-01	6.46E-01	1.23E+00	3.38E-01	4.78E-01	1.32E+00	2.17E-01	6.17E-02	3.95E-02	1.05E+00
(3g/day)	GM	1.83E-01	2.14E-03	2.34E-03	3.98E-03	5.36E-03	2.17E-03	2.38E-03	3.21E-03	1.57E-03	1.12E-03	3.59E-04	5.76E-04
Body weight	Max	1.49E+01	2.87E+00	4.94E+00	1.71E+01	2.02E+01	7.83E+00	1.02E+01	1.81E+01	3.42E+00	3.40E+00	1.59E+00	2.00E+01
(64.2 kg)	min	1.23E-01	7.48E-04	7.48E-04	1.31E-03	1.12E-03	1.12E-03	9.35E-04	5.14E-04	5.14E-04	9.35E-04	2.34E-04	4.67E-05
11~21 years old	Median	1.23E-01	7.48E-04	7.48E-04	1.31E-03	1.12E-03	1.12E-03	9.35E-04	5.14E-04	5.14E-04	9.35E-04	2.34E-04	4.67E-05
Application amount	Mean	5.48E-01	1.70E-01	2.52E-01	5.19E-01	9.88E-01	2.72E-01	3.84E-01	1.06E+00	1.74E-01	4.95E-02	3.17E-02	8.40E-01
(3g/day)	GM	1.47E-01	1.71E-03	1.88E-03	3.19E-03	4.30E-03	1.74E-03	1.91E-03	2.58E-03	1.26E-03	9.03E-04	2.88E-04	4.63E-04
Body weight	Max	1.20E+01	2.30E+00	3.96E+00	1.37E+01	1.62E+01	6.28E+00	8.16E+00	1.45E+01	2.74E+00	2.73E+00	1.27E+00	1.61E+01
(80 kg)	min	9.90E-02	6.00E-04	6.00E-04	1.05E-03	9.00E-04	9.00E-04	7.50E-04	4.13E-04	4.13E-04	7.50E-04	1.88E-04	3.75E-05
>21 years old	Median	9.90E-02	6.00E-04	6.00E-04	1.05E-03	9.00E-04	9.00E-04	7.50E-04	4.13E-04	4.13E-04	7.50E-04	1.88E-04	3.75E-05
Application amount	Mean	5.34E+00	1.66E+00	2.46E+00	5.06E+00	9.64E+00	2.65E+00	3.74E+00	1.03E+01	1.70E+00	4.83E-01	3.09E-01	8.19E+00
(9g/day)	GM	1.43E+00	1.67E-02	1.83E-02	3.11E-02	4.20E-02	1.70E-02	1.87E-02	2.51E-02	1.23E-02	8.81E-03	2.81E-03	4.51E-03

Body weight (24.6 kg)	Max	1.17E+02	2.24E+01	3.87E+01	1.34E+02	1.59E+02	6.13E+01	7.96E+01	1.42E+02	2.67E+01	2.66E+01	1.24E+01	1.57E+02
	min	9.66E-01	5.85E-03	5.85E-03	1.02E-02	8.78E-03	8.78E-03	7.32E-03	4.02E-03	4.02E-03	7.32E-03	1.83E-03	3.66E-04
2~11 years old	Median	9.66E-01	5.85E-03	5.85E-03	1.02E-02	8.78E-03	8.78E-03	7.32E-03	4.02E-03	4.02E-03	7.32E-03	1.83E-03	3.66E-04
Application amount (9g/day)	Mean	2.05E+00	6.35E-01	9.43E-01	1.94E+00	3.69E+00	1.02E+00	1.43E+00	3.96E+00	6.52E-01	1.85E-01	1.18E-01	3.14E+00
	GM	5.48E-01	6.41E-03	7.03E-03	1.19E-02	1.61E-02	6.52E-03	7.15E-03	9.64E-03	4.70E-03	3.37E-03	1.08E-03	1.73E-03
Body weight (64.2 kg)	Max	4.47E+01	8.60E+00	1.48E+01	5.12E+01	6.07E+01	2.35E+01	3.05E+01	5.43E+01	1.02E+01	1.02E+01	4.76E+00	6.01E+01
	min	3.70E-01	2.24E-03	2.24E-03	3.93E-03	3.36E-03	3.36E-03	2.80E-03	1.54E-03	1.54E-03	2.80E-03	7.01E-04	1.40E-04
11~21 years old	Median	3.70E-01	2.24E-03	2.24E-03	3.93E-03	3.36E-03	3.36E-03	2.80E-03	1.54E-03	1.54E-03	2.80E-03	7.01E-04	1.40E-04
Application amount (9g/day)	Mean	1.64E+00	5.09E-01	7.57E-01	1.56E+00	2.96E+00	8.15E-01	1.15E+00	3.18E+00	5.23E-01	1.48E-01	9.50E-02	2.52E+00
	GM	4.40E-01	5.14E-03	5.64E-03	9.58E-03	1.29E-02	5.23E-03	5.74E-03	7.73E-03	3.77E-03	2.71E-03	8.65E-04	1.39E-03
Body weight (80 kg)	Max	3.59E+01	6.90E+00	1.19E+01	4.11E+01	4.87E+01	1.89E+01	2.45E+01	4.36E+01	8.23E+00	8.19E+00	3.82E+00	4.83E+01
	min	2.97E-01	1.80E-03	1.80E-03	3.15E-03	2.70E-03	2.70E-03	2.25E-03	1.24E-03	1.24E-03	2.25E-03	5.63E-04	1.13E-04
>21 years old	Median	2.97E-01	1.80E-03	1.80E-03	3.15E-03	2.70E-03	2.70E-03	2.25E-03	1.24E-03	1.24E-03	2.25E-03	5.63E-04	1.13E-04
Application amount (13.5g/day)	Mean	8.02E+00	2.48E+00	3.69E+00	7.59E+00	1.45E+01	3.97E+00	5.61E+00	1.55E+01	2.55E+00	7.24E-01	4.63E-01	1.23E+01
	GM	2.15E+00	2.51E-02	2.75E-02	4.67E-02	6.30E-02	2.55E-02	2.80E-02	3.77E-02	1.84E-02	1.32E-02	4.22E-03	6.77E-03
Body weight (24.6 kg)	Max	1.75E+02	3.37E+01	5.80E+01	2.00E+02	2.38E+02	9.20E+01	1.19E+02	2.13E+02	4.01E+01	4.00E+01	1.87E+01	2.35E+02
	min	1.45E+00	8.78E-03	8.78E-03	1.54E-02	1.32E-02	1.32E-02	1.10E-02	6.04E-03	6.04E-03	1.10E-02	2.74E-03	5.49E-04

2~11 years old	Median	1.45E+00	8.78E-03	8.78E-03	1.54E-02	1.32E-02	1.32E-02	1.10E-02	6.04E-03	6.04E-03	1.10E-02	2.74E-03	5.49E-04
Application amount (13.5g/day)	Mean	3.07E+00	9.52E-01	1.41E+00	2.91E+00	5.54E+00	1.52E+00	2.15E+00	5.94E+00	9.78E-01	2.78E-01	1.78E-01	4.71E+00
	GM	8.23E-01	9.61E-03	1.05E-02	1.79E-02	2.41E-02	9.78E-03	1.07E-02	1.45E-02	7.05E-03	5.06E-03	1.62E-03	2.59E-03
Body weight (64.2 kg)	Max	6.71E+01	1.29E+01	2.22E+01	7.68E+01	9.11E+01	3.52E+01	4.57E+01	8.15E+01	1.54E+01	1.53E+01	7.15E+00	9.02E+01
	min	5.55E-01	3.36E-03	3.36E-03	5.89E-03	5.05E-03	5.05E-03	4.21E-03	2.31E-03	2.31E-03	4.21E-03	1.05E-03	2.10E-04
11~21 years old	Median	5.55E-01	3.36E-03	3.36E-03	5.89E-03	5.05E-03	5.05E-03	4.21E-03	2.31E-03	2.31E-03	4.21E-03	1.05E-03	2.10E-04
Application amount (13.5g/day)	Mean	2.47E+00	7.64E-01	1.14E+00	2.33E+00	4.44E+00	1.22E+00	1.73E+00	4.76E+00	7.85E-01	2.23E-01	1.42E-01	3.78E+00
	GM	6.60E-01	7.72E-03	8.46E-03	1.44E-02	1.94E-02	7.85E-03	8.61E-03	1.16E-02	5.66E-03	4.06E-03	1.30E-03	2.08E-03
Body weight (80 kg)	Max	5.38E+01	1.03E+01	1.78E+01	6.16E+01	7.31E+01	2.83E+01	3.67E+01	6.54E+01	1.23E+01	1.23E+01	5.74E+00	7.24E+01
	min	4.46E-01	2.70E-03	2.70E-03	4.73E-03	4.05E-03	4.05E-03	3.38E-03	1.86E-03	1.86E-03	3.38E-03	8.44E-04	1.69E-04
>21 years old	Median	4.46E-01	2.70E-03	2.70E-03	4.73E-03	4.05E-03	4.05E-03	3.38E-03	1.86E-03	1.86E-03	3.38E-03	8.44E-04	1.69E-04

GM: geometric mean

* samples which were non detected were calculated with the value of detection limit divided by the square root of 2.
